# Spontaneous Ocular Scanning of Visual Symmetry Is Similar During Classification and Evaluation Tasks

**DOI:** 10.1177/2041669520946356

**Published:** 2020-10-12

**Authors:** Alexis D. J. Makin, Ellen Poliakoff, Giulia Rampone, Marco Bertamini

**Affiliations:** Department of Psychological Sciences, University of Liverpool, United Kingdom; Division of Neuroscience and Experimental Psychology, School of Biological Sciences, Faculty of Biological, Medical and Human Sciences, Manchester Academic Health Science Centre, The University of Manchester, United Kingdom; Department of Psychological Sciences, University of Liverpool, United Kingdom

**Keywords:** aesthetics, reflection, symmetry, saccades, eye movements

## Abstract

Visual symmetry perception and symmetry preference have been studied extensively. However, less is known about how people spontaneously scan symmetrical stimuli with their eyes. We thus examined spontaneous saccadic eye movements when participants (*N* = 20) observed patterns with horizontal or vertical mirror reflection. We found that participants tend to make saccades along the axis of reflection and that this oculomotor behaviour was similar during objective classification and subjective evaluation tasks. The axis-scanning behaviour generates a dynamic sequence of novel symmetrical images from a single static stimulus. This could aid symmetry perception and evaluation by enhancing the neural response to symmetry.

Symmetry preference and symmetry perception are important topics in visual aesthetics and visual science. Ramachandran and Hirstein (1999) noted that artists from all cultures have celebrated the aesthetic value of symmetry. This could have evolutionary origins: Many animals are instinctively attracted to health-signalling phenotypic symmetry in potential mates (Grammer et al., 2003). Likewise, humans are attracted to symmetrical faces (Little et al., 2011) and bodies (Bertamini et al., 2013). Abstract symmetry is also aesthetically pleasing (Jacobsen & Höfel, 2002; Makin et al., 2012; Rentschler et al., 1999). In their review of empirical aesthetics, Palmer et al. (2013) surmised that abstract symmetry preference is robust, although there are interesting individual differences (Leder et al., 2019).

The neural basis of symmetry perception has also been studied (for a review, see Bertamini et al., 2018). Symmetry activates a network of extrastriate visual areas (including the Lateral Occipital Complex) but not V1 or V2 (Sasaki et al., 2005). This extrastriate symmetry response generates an event-related potential called the sustained posterior negativity (SPN; Jacobsen & Höfel, 2003; Makin et al., 2016). The SPN is similar when participants are classifying symmetry or evaluating beauty (Höfel & Jacobsen, 2007).

During normal behaviour, the eyes actively explore the world with a series of purposeful fixations and saccades, bringing objects of interest onto the fovea for detailed visual examination (Findlay & Gilchrist, 2001; Itti & Koch, 2001). However, little is known about how people spontaneously scan symmetry with their eyes. After all, electroencephalogram studies usually suppress eye movements with task instructions and by using small stimuli in the foveal region (Jacobsen & Höfel, 2003). Meanwhile, most symmetry preference studies do not measure eye movements. Some work has documented preferential looking towards symmetry in infants (Bornstein et al., 1981); however, this does not tell us how people scan the symmetrical image itself. Mühlenbeck et al. (2016) presented pairs of symmetrical and asymmetrical images for 3 seconds and found that adolescents (from industrial and non-industrial societies) fixated the symmetrical option for around 180 ms longer on average. This replicates preferential looking towards symmetry in different populations but, again, does not tell us about internal scanning of symmetrical images.

It is tempting to think that people must saccade back and forth across the axis to find spatial correspondences. However, there are three lines of evidence against this. First, symmetry can be detected within 50 milliseconds and thus within a single fixation (Carmody et al., 1977). Second, visual symmetry generates a brain response even when participants are forced to fixate (Makin et al., 2016). Third, gaze is reflexively attracted to symmetrical objects in the periphery, so symmetry detection can *precede* internal ocular scanning of the symmetrical halves (Kootstra et al., 2011).

In the current study, we examined spontaneous ocular scanning of vertical and horizontal reflection. We used checkerboard patterns comparable to those used in previous work (Mancini et al., 2005; Royer, 1981). We used relatively large 13.5° × 13.5° checkerboards partly because we wanted any exploratory saccades to be easily detectable with the eye tracker and partly because we wanted high-contrast symmetrical substructures to be visible in the periphery so eye movements were not essential for accurate symmetry discrimination.

We compared saccades during objective classification (symmetry/random) and subjective evaluation (ugly to beautiful). This provides insights into whether the stimuli or task are responsible for observed oculomotor behaviour. This is an important question because aesthetic evaluation may have a distinct oculomotor profile. Berlyne (1971) suggested people can engage in either diverse or specific modes of aesthetic exploration. These modes could result in different saccadic eye movements, for instance, when viewing Mondrian paintings (Plumhoff & Schirillo, 2009).

Our experiment follows several others that have used different tasks or stimuli. Meso et al. (2016) compared saccades during free viewing and orientation discrimination tasks. Their stimuli were black and white dot patterns (23.4° diameter with 512 dots). The dots were either randomly arranged or reflected across an axis with vertical, horizontal, or oblique orientation. Mean eye position remained approximately in the centre. However, there were low-amplitude saccades directed along the axes. The saccades were similar during free viewing and orientation discrimination tasks.

As reviewed in Locher and Nodine (1989), some early studies have found that people often fixate on just one side of symmetrical patterns after the first few exploratory saccades. However, these early studies used large shapes with no central symmetrical texture, so this is likely to be a special case. Indeed, Mühlenbeck et al. (2016) found that humans typically fixated the centre of symmetrical patterns after initial exploratory saccades, while orangutans scanned the whole pattern and its surroundings.

Locher et al. (1993) presented 20° × 20° dot patterns for 20-second intervals. These were vertical reflection, horizontal reflection, or random. Participants judged complexity, pleasingness, or searched for small E-shaped targets (which were not actually present). In complexity and pleasingness tasks, participants tended to fixate vertical and horizontal axes. In the impossible search task, fixations were more evenly distributed across the pattern.

The findings of Locher et al. (1993) and Meso et al. (2016) suggest that our participants will direct saccades along the axes in both tasks. However, this is not a foregone conclusion. Meso et al. (2016) did not examine aesthetic evaluation. Meanwhile, our study differs from Locher et al. (1993) in three crucial ways. First, we examined all saccades over just 3 seconds rather than average fixation locations over 20 seconds. This yields much higher resolution oculomotor behaviour. Second, we compared symmetry discrimination with aesthetic evaluation, while Locher et al. (1993) compared complexity discrimination with aesthetic evaluation. Third Locher et al. (1993) used dot patterns where elements were not visible in the periphery, while we used checkerboards where symmetrical substructures were visible in the periphery. Therefore, our experiment is a necessary extension of Locher et al. (1993) and Meso et al. (2016).

One possibility is that top-down task influences change over time. While the initial saccades could be stimulus driven, some later saccades could be specific to aesthetic evaluation. This is found in other work with art stimuli: Reliable aesthetic judgements about paintings can be made in a single fixation; however, a second stage of exploration can ensure if paintings are sufficiently interesting (Locher, 2015). We therefore separated our analysis into three time windows (0–1 seconds, 1–2 seconds, and 2–3 seconds) to explore the time course of oculomotor behaviour.

## Method

### Participants

Twenty participants were included (aged 18–27 years, mean age = 23.5 years, 5 male, and 1 left-handed). All participants had normal or corrected-to-normal vision. The experiment had local ethics committee approval. The participants were undergraduate and postgraduate students at the University of Manchester.

### Apparatus

The experiment was programmed in Python using PsychoPy 1.82 libraries (Peirce, 2007). The experiments are available on *open science framework*, along with raw data, preprocessing scripts, and online materials (https://osf.io/br583/). Participants sat approximately 75 cm away from a 53 × 30 cm 60 HZ LCD screen. The eye tracker was calibrated using a nine-point calibration sequence. Eye position was recorded with an EyeLink 1000Plus eye tracker at 500 Hz. Data were recorded for 4 seconds on each trial (–1 to + 3 seconds around pattern onset). We alternated left and right eye recording between participants.

### Stimuli

**Figure 1.**
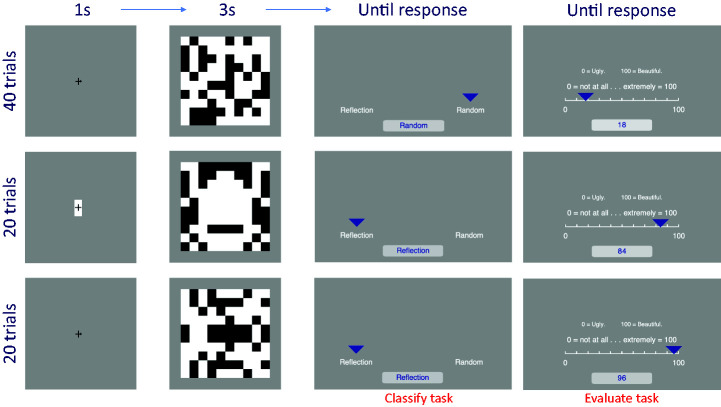
Trial Structure in Classify and Evaluate Tasks.

Patterns were a 10 × 10 checkerboard with 40 black and 60 white checks (Figure 1). The checkerboard was approximately 13.5° × 13.5° of visual angle. On each trial, a novel checkerboard was created using the same algorithm. On random trials, both sides were constructed independently. On reflection trials, one half was mirrored.

### Procedure

There were 80 trials in the Classify task and 80 trials in the Evaluate task. Of these, 40 trials were random, 20 were vertical reflection, and 20 were horizontal reflection (Table 1). Each trial began with a fixation cross presented for 1 second (Figure 1). For the following 3 seconds, the pattern was on the screen (as in Meso et al., 2016). In the Classify task, participants used the mouse to classify the patterns as “reflection” or “random” (the left–right positioning of these options was balanced between trials). In the Evaluate task, they used the mouse to rate the patterns on a 0 to 100 analogue scale. The extremes of the scale were labelled as “Ugly” (left) and “Beautiful” (right). Task order was counterbalanced, so half of the participants completed the Classify task first, and the other half completed the Evaluate task first.

**Table 1.** Saccade Descriptive Statistics.

Regularity	Task	Interval (s)	*N* participants	*N* trials	Recorded data (s)	*N* saccades	*N* eligible saccades	% used	Saccade rate (Hz)
Random	Classify	3	20	40	2,400	6,182	6,155	99.6	2.56
Random	Evaluate	3	20	40	2,400	5,841	5,791	99.1	2.41
Vertical	Classify	3	20	20	1,200	3,158	3,131	99.1	2.61
Vertical	Evaluate	3	20	20	1,200	3,102	3,084	99.4	2.57
Horizontal	Classify	3	20	20	1,200	3,228	3,187	98.7	2.66
Horizontal	Evaluate	3	20	20	1,200	3,059	3,041	99.4	2.53

The tasks were explained by the experimenter (A. D. J. M.), and the participants were presented with onscreen instructions. In the Classify task, instructions read, “please indicate if the patterns were symmetric or random.” In the Evaluate task, instructions read, “please indicate how much you like the patterns.” There was no practice block because the tasks were very intuitive. The experimenter was in the same lab area operating the eye tracker and could help if participants were uncertain about the tasks. The experimental session lasted approximately 30 minutes.

### Saccade Analysis

**Figure 2.**
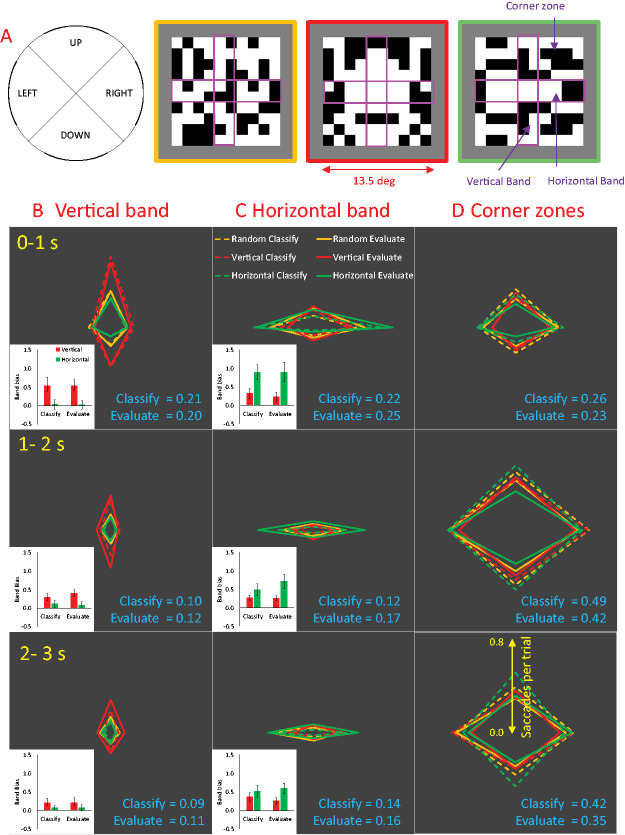
Saccade Rate. (A) Diagram of vertical bands, horizontal bands, and corner zones superimposed on example patterns. (B) There were more saccades in the vertical band when the axis of reflection was vertical (red diamonds). Saccade rate in the vertical band decreased across the three intervals (rows). (C) There were more saccades in the horizontal band when the axis of reflection was horizontal (green diamonds). Saccade rate in the horizontal band decreased across the three intervals. Insets show band bias (e.g., diamond elongation) in the reflection conditions. Error bars = 95% CI. (D) The number of saccades which either started or ended outside these bands, in the corner zones, increased across the three intervals. Mean saccade rates in Classify and Evaluate tasks are shown in blue text (saccades per trial).

Saccades where amplitude exceeded 0.5° were identified automatically with *Dataviewer* software (version 2.3.0; SR Research). Saccades were then classed as those which occurred in (a) the *vertical band*, (b) the *horizontal band*, or (c) those which started and/or ended outside these bands in the *corner zones* (Figure 2A). Saccades direction was classed as upwards, downwards, leftwards, or rightwards, depending on saccade angle output. Saccade rate and amplitude were computed during the first, second, and third intervals of the 3-second presentation.

Table 1 shows descriptive statistics of the saccades in six conditions: 2 Task (classify, evaluate) × 3 Stimuli (random, vertical, horizontal). It can be seen that eligible saccade rate was consistent across the conditions (at 2.41 to 2.66 Hz).

For repeated measures analysis of variance, the Greenhouse–Geisser correction factor was used when the assumption of sphericity was violated (Mauchly’s test *p* < .05). We report the largest and most theoretically interesting effects here. Full analysis of variance is available on open science framework (https://osf.io/br583/). Given that we report many effects and interactions, false positive rate is inflated. Corrections for familywise error rate would thus abolish the weaker effects. However, all the effects that we theoretically interpret in the article were very substantial (*p* < .001, effect size > 0.5) and would survive correction for familywise error rate.

## Results

### Behavioural Ratings

In the Classify task, performance was near ceiling (mean correct random = 98%, vertical 94%, horizontal 95%). In the Evaluate task, there was a significant effect of Regularity on mean preference ratings—random = 31.33/100, vertical = 62.21, horizontal = 57.65, *F*(1.269, 24.104) = 49.623, *p* < .001, η_p_^2^ = 0.723. Participants preferred both vertical and horizontal reflection to random, *t*(19) = 7.721, *p* < .001, *t*(19) = 6.823, *p* < .001. These effects were found in 19/20 participants. Participants also preferred vertical reflection to horizontal reflection, *t*(19) = 2.765, *p* = .012, (15/20 participants).

### Saccade Rate

Figure 2 shows saccade rate from three time intervals (0–1 seconds, 1–2 seconds, and 2–3 seconds post stimulus onset) and three spatial regions (vertical band, horizontal band, and corner zones). It can be seen that participants tended to orientate saccades along the vertical or horizontal axis of reflection when it was present in the stimuli. However, this oculomotor behaviour became less frequent towards the end of the trial. Conversely, saccades touching the corner zones became more frequent towards the end of the trial. These saccades rate metrics were similar in the Classify and Evaluate tasks.

Figure 2B shows the distribution of saccades that fell within the *vertical band*. The radar plot shows saccade rate in each of the four directions (the more eccentric the vertex of the diamond, the more saccades per trial in that direction). It can be seen that there were more upward and downward saccades when the axis of reflection was vertical (elongated red diamonds) than when the axis of reflection was horizontal (shorter green diamonds). The number of saccades falling within the vertical band decreased towards the end of the trial (smaller diamonds in rows 2 and 3).

Figure 2C shows the distribution of saccades that fell within the *horizontal band*. There were more leftward and rightward saccades when the axis of reflection was horizontal (green diamonds) than vertical (red diamonds). The number of saccades falling within the horizontal band also decreased towards the end of the trial.

Figure 2D shows the distribution of saccades that fell outside these bands that either started or ended in the *corner zones*. These saccades became more frequent during the middle and end of the trials. Corner zone saccade rate was similar for random, vertical, and horizontal symmetry.

We first analysed saccade rate in the three Zones (vertical band, horizontal band, and corners) and the three Intervals (0–1 seconds, 1–2 seconds, and 2–3 seconds) and two Tasks (Classify and Evaluate), collapsing across factors Regularity (random, vertical, and horizontal) and Saccade Direction (up, down, left, and right). This analysis confirms impressions about the *area* of the diamonds in Figure 2B to D rather than their shape. There was no main effect of Task (*F* < 1). The strongest effect was a Zone × Interval interaction, *F*(2.549, 48.439) = 64.497, *p* < .001, η_p_^2^ = 0.772, which did not interact with Task, *F*(1.891, 35.933) = 2.085, *p* = .141. Saccade rate decreased over time in the vertical band (the strongest polynomial contrast was linear), *F*(1, 19) = 50.187, *p* < .001, η^2^ = 0.725. Saccade rate also decreased over time in the horizontal band (linear contrast), *F*(1,19) = 27.686, *p* < .001, η^2^ = 0.593. Conversely, saccades to or from the corner zones were most frequent during the central interval (strongest contrast was quadratic), *F*(1,19) = 102.976, *p* < .001, η^2^ = 0.844.

To examine the effect of axis orientation on saccade direction, we computed a metric called “band bias” from each participant and condition: *band bias = (parallel saccades – orthogonal saccades)/total trials.* Band bias indexes how many more band-parallel saccades than band-orthogonal saccades happened per trial. We expected band bias to be uniformly greater than zero because bands already filter saccades of the same orientation. More importantly, we tested whether band bias was enhanced by a band-parallel axis (vertical, horizontal) and whether this was comparable in Classify and Evaluate tasks. We did not include random trials in this analysis. Band bias is shown in white insets in 2B and C.

The expected effect of axis orientation on band bias was confirmed by a significant Band × Axis interaction, *F*(1, 19) = 52.539, *p* < .001, η_p_^2^ = 0.734. This Band × Axis interaction was strongest in the first interval, *F*(1, 19) = 49.282, *p* < .001, η_p_^2^ = 0.722, (effect present in all 20 participants). It was also strong in the second interval, *F*(1, 19) = 44.689, *p* < .001, η_p_^2^ = 0.702, (effect present in 19/20 participants), but weaker in the third interval, *F*(1, 19) = 19.581, *p* < .001, η_p_^2^ = 0.508, (18/20 participants). This reduction over time was confirmed by a significant Band × Axis × Interval interaction, *F*(2, 38) = 22.004, *p* < .001, η_p_^2^ = 0.537. This three-way interaction was not further modulated by Task (*F* < 1), suggesting oculomotor exploration of the axes was similar in both tasks.

### Saccade Amplitude

**Figure 3.**
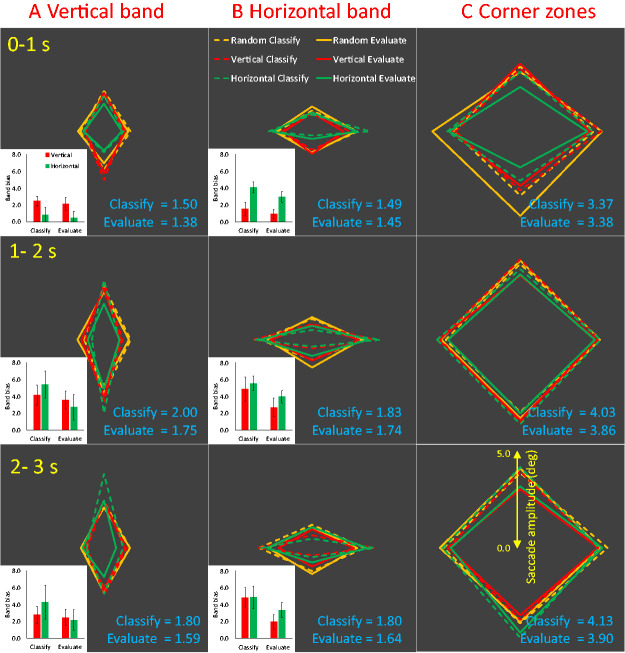
Saccade Amplitude. The conventions are the same as Figure 2 but show mean saccade amplitude (in °) rather than saccade rate. The mean saccade amplitude in Classify and Evaluate tasks are shown in blue text in each panel (in °).

Figure 3 illustrates saccade amplitude. The trend for higher amplitude saccades in the Classify task was not significant, *F*(1, 19) = 2.546, *p* = .127. Saccades to or from the corner zones were higher amplitude than those that fell exclusively within the horizontal and vertical bands, *F*(1.075, 20.418) = 69.372, *p* < .001, η_p_^2^ = 0.785. Saccade amplitude peaked in the central interval (the strongest polynomial contrast was quadratic), *F*(1, 19) = 34.589, *p* < .001, η_p_^2^ = 0.645.

Unsurprisingly, vertical saccade amplitude was greater in the vertical band, and horizontal saccade amplitude was greater in the horizontal band. Interestingly, this band bias was enhanced by axis orientation *but only in the first interval*. This means that only first few saccades on each trial were extended along axes. The crucial Band × Axis interaction was present the first interval, *F*(1, 19) = 55.701, *p* < .001, η_p_^2^ =0.746, but not in the second, *F*(1, 19) = 1.010, *p* = .328, or third (*F* < 1). Although the Band × Axis interaction was more persistent in the Evaluate task, the Band × Axis × Interval interaction, *F*(2, 38) = 11.625, *p* < .001, η_p_^2^ = 0.380, was not itself modulated by Task, *F*(2, 38) = 2.626, *p* = .085.

Finally, we examined the standard deviation (SD) of saccade amplitudes across trials. Although saccade amplitude is sometimes more variable during aesthetic evaluation (Plumhoff & Schirillo, 2009), there was no evidence for this in our experiment (main effect of Task on SD; *F* < 1). Of the 216 conditions shown in Figure 3, those with greater mean saccade amplitude also had greater SD (*r* = .95, *p* < .001). Consequently, most observed amplitude effects in Figure 3 were also evident on SD (https://osf.io/br583/).

## Discussion

Our results replicated Meso et al. (2016) with different stimuli and tasks. Relatively small saccades (mean amplitude approximately 2.4°, or 2 checkerboard elements) were disproportionately directed along the axes of reflection, particularly during the early part of the trials. Later, participants made more high amplitude saccades into the corner zones (mean amplitude ∼3.8°).

This oculomotor behaviour was broadly similar in objective classification and subjective evaluation tasks. This parallels earlier work from Locher et al. (1993), who found people preferentially fixate horizontal and vertical axes, both when judging complexity and pleasingness (although not during an impossible visual search task). Therefore, there was no distinct oculomotor signature of aesthetic evaluation, even though our participants had the typical preference for symmetry (Jacobsen & Höfel, 2002; Makin et al., 2012; Rentschler et al., 1999). Aesthetic evaluation of paintings may sometimes involve specific modes of oculomotor exploration (Locher, 2015; Plumhoff & Schirillo, 2009); however, this was not apparent with our simple checkerboards. In summary, saccades were determined by bottom-up stimulus factors more than top-down task factors. With other Stimuli × Task combinations, there would likely be a more complicated interplay between the two (Locher et al., 1993). We also note that members of other cultures, and particularly other species, may not scan symmetry in the same way (Mühlenbeck et al., 2016).

Let us consider why participants scan the axis of reflection with exploratory eye movements when symmetry can already be discriminated during a single fixation (Carmody et al., 1977). By moving the eyes along the axis, the visual cortex receives several alternative images of the symmetry in quick succession. It is known that rapid presentation of different symmetrical exemplars enhances discrimination performance (Sharman & Gheorghiu, 2017) and enhances SPN amplitude (Bertamini et al., 2019). By moving the eyes along the axis, the same dynamic enhancement effect could be achieved from *static* stimuli.

While symmetry can be discriminated within 50 milliseconds, performance on psychophysical tasks improves when stimulus duration is increased up to 1,000 milliseconds (Tyler et al., 1995). This improvement with presentation time could be due to the putative dynamic oculomotor enhancement effect. Furthermore, even if fixation is required, covert shifts of attention could achieve a similar enhancement.

The current work also tells us something about the time course of symmetry perception and evaluation. Axis scanning was concentrated in the first second, and saccades were elongated only along the axes in the first second. This suggests all perceptual advantages gained by axis scanning can be obtained within 1 second, so participants are free to examine novel substructures in the corner zones thereafter.

There is some similarity between the observed axis-scanning behaviour and hypothetical symmetry perception mechanisms. For instance, the *bootstrapping model* suggests symmetry is discovered by iterative integration of symmetry information from successive points along the axis (Wagemans et al., 1993). However, the model does not explicitly suggest a role for saccades, and the iterative bootstrapping operation might happen rapidly within a single fixation. *Filter models* propose symmetry perception involves low-pass filtering to extract axis-orthogonal blobs, followed by estimation of blob alignment (Dakin & Watt, 1994; Rainville & Kingdom, 2000). Estimation of blob alignment might involve saccades from the centre of one blob to the next, although blob alignment estimation, such as bootstrapping, may happen within a single fixation (Dakin & Hess, 1997). While these links are speculative, the fact that people spontaneously saccade along the axis is an empirical observation which could constrain future models of symmetry perception.

There are four limitations with this study. First, participants may have anticipated the response screen during the presentation interval, and this could have differentially biased their eye movements in Classify and Evaluate tasks. However, given that saccades were more influenced by bottom-up stimulus factors than top-down task factors, we do not think this complicates our conclusions. Second, our decision to block rather than interleave the Classify and Evaluate trials could have been consequential. However, blocking is likely to have maximized any existing task effects, and we still found intertask similarity nevertheless. Third, we cannot be sure that conclusions generalize beyond checkerboard stimuli. However, given that comparable axis scanning with dot patterns was found by Locher et al. (1993) and Meso et al. (2016), this is likely a general phenomenon. Future work could measure scanning of rotational symmetry or glass patterns and vary stimulus size systematically. Fourth, there were some unexpected weaker effects reported in Supplemental Material. However, some of these may be false positives. Importantly, the bottom-up effects driven by axis orientation were much more robust than any top-down effects driven by task.

## Conclusion

We have shown that people spontaneously make saccades along the axis of reflection. This oculomotor behaviour replicates previous work, and we found that the same behaviour occurs during aesthetic evaluation. It could be that the extrastriate symmetry response is boosted by saccade-driven updates of the retinal image, and this aids both objective and subjective judgements.
